# Robust Superconductivity in Infinite‐Layer Nickelates

**DOI:** 10.1002/advs.202305252

**Published:** 2024-04-29

**Authors:** Minghui Xu, Yan Zhao, Yu Chen, Xiang Ding, Huaqian Leng, Zheng Hu, Xiaoqiang Wu, Jiabao Yi, Xiaojiang Yu, Mark B.H. Breese, Shibo Xi, Mengsha Li, Liang Qiao

**Affiliations:** ^1^ School of Physics University of Electronic Science and Technology of China Chengdu 610054 China; ^2^ Center for Microscopy and Analysis Nanjing University of Aeronautics and Astronautics Nanjing 211100 China; ^3^ Institute for Advanced Study Chengdu University Chengdu 610106 China; ^4^ Global Innovative Centre for Advanced Nanomaterials, School of Engineering The University of Newcastle Callaghan NSW 2308 Australia; ^5^ Singapore Synchrotron Light Source National University of Singapore Singapore 117603 Singapore

**Keywords:** nickelates, reversible, superconductor, topological relationships

## Abstract

The recent discovery of nickelate superconductivity represents an important step toward understanding the four‐decade mastery of unconventional high‐temperature superconductivity. However, the synthesis of the infinite‐layer nickelate superconductors shows great challenges. Particularly, surface capping layers are usually unitized to facilitate the sample synthesis. This leads to an important question whether nickelate superconductors with *d^9^* configuration and ultralow valence of Ni^1+^ are in metastable state and whether nickelate superconductivity can be robust? In this work, a series of redox cycling experiments are performed across the phase transition between perovskite Nd_0.8_Sr_0.2_NiO_3_ and infinite‐layer Nd_0.8_Sr_0.2_NiO_2_. The infinite‐layer Nd_0.8_Sr_0.2_NiO_2_ is quite robust in the redox environment and can survive the cycling experiments with unchanged crystallographic quality. However, as the cycling number goes on, the perovskite Nd_0.8_Sr_0.2_NiO_3_ shows structural degradation, suggesting stability of nickelate superconductivity is not restricted by the ultralow valence of Ni^1+^, but by the quality of its perovskite precursor. The observed robustness of infinite‐layer Nd_0.8_Sr_0.2_NiO_2_ up to ten redox cycles further indicates that if an ideal high‐quality perovskite precursor can be obtained, infinite‐layer nickelate superconductivity can be very stable and sustainable under environmental conditions. This work provides important implications for potential device applications for nickelate superconductors.

## Graphical abstract

The infinite‐layer structure is achieved by deintercalation of apical oxygens of perovskite nickelates, forming alternating planer [NiO_2_] layers and [Nd_0.8_Sr_0.2_] space layers. Therefore, it is important to give a comprehensive understanding of oxygen evolution in space layers and its impact on nickelate superconductivity. A reversible transition is demonstrated between the perovskite Nd_0.8_Sr_0.2_NiO_3_ and the infinite‐layer Nd_0.8_Sr_0.2_NiO_2_ thin films without losing their structural framework and topological relationships. The superconductivity in Nd_0.8_Sr_0.2_NiO_2_ still exists even for ten times of “deintercalation–intercalation” cycles, indicating the robustness of the infinite‐layer structural frame.

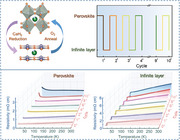

## 1 Introduction

Oxygen stoichiometry is crucial for perovskite transition metal oxides, as it directly determines both coordination and formal valence charge of transition metal cations, thus playing critical roles in regulating the electronic, magnetic, and related functionalities. Yet, precision control of oxygen concentration in perovskites is a challenging task. Especially, the oxygen‐deficient nickelate RNiO_(3‐δ)_, has attracted significant research attention because of the drastic changes in its crystal structure and physical properties in response to oxygen content variation.^[^ ^1^, ^2^ ^]^

In fact, the study of oxygen‐deficient nickelate can be dated back to the 1980s, when the polycrystalline perovskite LaNiO_3_ was reduced to infinite‐layer LaNiO_2_ using hydrogen as a reducing agent.^[^ ^3^, ^4^ ^]^ Later on, effective reduction using metal hydrides was achieved at lower temperatures, and the technique was further extended to epitaxial nickelate films, with the first demonstration of epitaxial perovskite LaNiO_3_(001) films into infinite‐layer LaNiO_2_ films using CaH_2_ in 2009.^[^ ^5^, ^6^, ^7^, ^8^ ^]^ Infinite‐layer LaNiO_2_ has square–planar NiO_4_ coordination with monovalent Ni^+^ *d* ^9^ configuration, resembling the square–plane‐coordinated Cu^2+^ in cuprates superconductors. In 2019, Li et al.^[^ ^9^ ^]^ reported the ground‐breaking discovery that hole‐doped infinite‐layer nickelate Nd_0.8_Sr_0.2_NiO_2_ films can be superconducting, opening a new research era for high‐temperature superconductivity. Although nickelates share some similarities with cuprates, such as 2D square lattice, isoelectronic 3𝑑^9^ valence state, superconducting domes in the phase diagram, etc.,^[^ ^10^, ^11^, ^12^, ^13^ ^]^ there are also obvious differences. For example, nickelate shows a weak Mott Hubbard feature,^[^ ^14^ ^]^ while cuprate belongs to the charge‐transfer insulator system.^[^ ^15^, ^16^ ^]^ Furthermore, to mimic the *d^9^* configuration of undoped cuprates, nickelates require chemical reduction to stabilize the infinite‐layer structure and achieve an ultralow valence state of Ni (Ni^1+^).

Recently, substantial experimental progress has been made on nickel‐based superconductivity, including in material growth, electronic structure, and physical properties.^[^ ^9^, ^12^, ^13^, ^17^, ^18^, ^19^, ^20^, ^21^, ^22^, ^23^, ^24^, ^25^, ^26^, ^27^, ^28^, ^29^, ^30^ ^]^ In particular, researchers investigated the relationship between nickelate doping concentration and superconducting transition temperature (*T*_c_) and established superconducting phase diagram,^[^ ^12^, ^13^ ^]^ antiferromagnetic correlations,^[^ ^31^, ^32^ ^]^ charge density waves (CDW) in the parent nickelates,^[^ ^33^, ^34^ ^]^ etc., however, this raises the important question of whether infinite‐layer nickelate superconductors with *d^9^* conformation and Ni^1+^ anomalously low nickel valence state are in a sub‐stable state and whether nickelate superconductivity is robust? On the other hand, over the past three decades, it has been well‐known that the synthesis of nickel‐based materials, particularly perovskite nickelates is greatly limited by oxygen deficiency. Therefore, the physical properties of nickelates are very sensitive to oxygen nonstoichiometry, subtle loss of oxygen can dramatically change the electronic, transport, and magnetic properties. Currently, the research field of superconducting nickelates is still in its infancy stage. Hence, it is important to fabricate high‐quality films and to fully understand the correlation of oxygen and nickelates superconductivity. In this consideration, superconducting infinite‐layer nickelates are an excellent platform for the study of reversible redox reactions and related physical changes, as they reflect the key role of oxygen content by controlling the oxygen atom state and thus regulating the oxidation state of nickel.

In this work, we demonstrated a reversible transition between the perovskite Nd_0.8_Sr_0.2_NiO_3_ and the infinite‐layer Nd_0.8_Sr_0.2_NiO_2_ thin films without losing their structural framework and topological relationships. The structural and physical properties of nickelates were systematically investigated by successively deintercalation and intercalation of the apical oxygens in sixfold coordination of Ni ions. It is found that the infinite‐layer Nd_0.8_Sr_0.2_NiO_2_ is generally robust in the redox environment and can survive the cycling experiments with unchanged crystallographic quality as evidenced by X‐ray diffraction (XRD), X‐ray absorption spectroscopy (XAS), and Hall Effect transport data. The superconductivity in Nd_0.8_Sr_0.2_NiO_2_ still exists even for ten times of “deintercalation–intercalation” cycles, indicating the robustness of the infinite‐layer structural frame. Further, we discuss in detail the effect of the competition between the perovskite Nd_0.8_Sr_0.2_NiO_3_ and Ruddlesden–Popper defect phases (RP‐type phases) on the subsequent formation of Nd_0.8_Sr_0.2_NiO_2_ superconducting infinite‐layer during the reversible modulation process.

## 2 Results and Discussion

**Figure 1:**
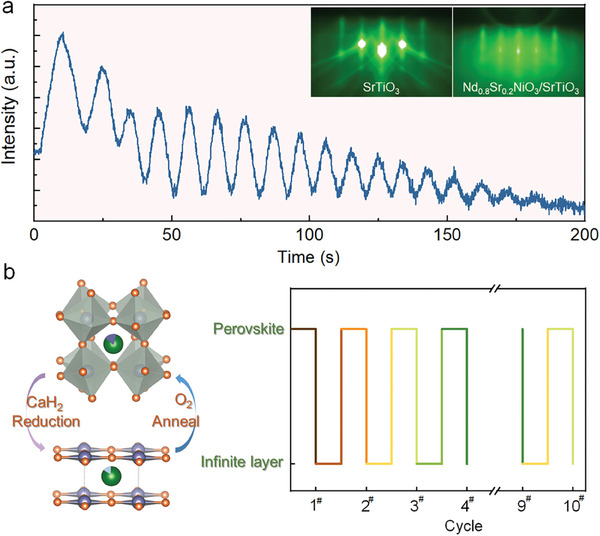
a) Typical RHEED intensity oscillations of Nd_0.8_Sr_0.2_NiO_3_ films grown along the SrTiO_3_ (100) azimuthal direction. The inset of which is the RHEED spot of the film before and after growth. The oscillations show the layer‐by‐layer growth of the film at the initial stage of growth, and the layer‐by‐layer growth gradually disappears as the film thickens. b) Oxygen atom trajectories in the crystal structure of the nickelates during topological reduction and the mechanism of the redox cycling experiments are plotted.

Fully strained nickelate Nd_0.8_Sr_0.2_NiO_3_ films with a step surface were grown by pulsed laser deposition (PLD) with layer‐by‐layer mode on single‐ended SrTiO_3_(001) substrates and monitored using reflected high‐energy electron diffraction (RHEED). **Figure** **1**a shows the typical RHEED intensity oscillations during the growth of perovskite nickelate Nd_0.8_Sr_0.2_NiO_3_ on SrTiO_3_, with ≈45 unit‐cells (≈17 nm) of deposited Nd_0.8_Sr_0.2_NiO_3_, as determined by the RHEED oscillation results. In addition, the inset of Figure 1a shows the RHEED spot pattern before and after deposition without observing transmission spots or any indications of precipitation, indicating uniform film growth and a smooth surface. Figure 1b depicts the lattice structure model of the oxygen atom trajectory during the reversible phase transition; and a schematic diagram of the reversible cycle between the two phases, where ten reversible cycles phase transitions between perovskite and infinite‐layer structures were performed in this study.

**Figure 2:**
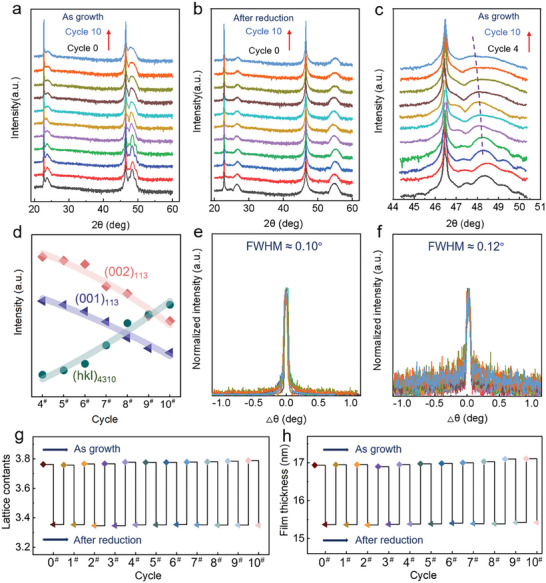
(Color online) Structural characteristics of doped nickelate films during reversible cycling transition. a) X θ−2θ symmetric scans of 15‐nm‐thick Nd_0.8_Sr_0.2_NiO_3_ and b) Nd_0.8_Sr_0.2_NiO_2_. c) Enlarged view of the Nd_0.8_Sr_0.2_NiO_3_ (002) diffraction peak of the perovskite structure in Figure 2a. d) Intensity ratios of Nd_0.8_Sr_0.2_NiO_3_, Nd_0.8_Sr_0.2_NiO_2_, and Nd_4_Ni_3_O_10_ peaks as a function of cycle (starts at cycle 4). e) Rocking curve scans across the (002) diffraction peak for the Nd_0.8_Sr_0.2_NiO_3_ and f) the superconducting Nd_0.8_Sr_0.2_NiO_2_ thin films. g) Variation curves of lattice constants and h) film thickness in redox cycling experiments for two structural nickelates.

The results of 2*θ−θ* X‐ray diffraction measurements with Cu Kα radiation are shown in **Figure** **2** for the as‐grown Nd_0.8_Sr_0.2_NiO_3_ films grown on SrTiO_3_ (001) substrates, where the (00l) reflections from the Nd_0.8_Sr_0.2_NiO_3_ film and the SrTiO_3_ (001) substrate are visible in the pattern and clearly show the structural characterization of the reversible transition of nickelate films from the perovskite to the infinite layer structure. All reversible cycles of the perovskite structured Nd_0.8_Sr_0.2_NiO_3_ films exhibited visible similar Bragg peaks in the substrate (002) indicating epitaxial growth shown in Figure 2a. The presence of fringe at the edge around the prominent cycle 0 (pristine) Nd_0.8_Sr_0.2_NiO_3_ film peak in the symmetric *θ*−2*θ* scan indicates the high quality of the epitaxial film. The deintercalation of octahedral apical oxygen atoms of the original perovskite Nd_0.8_Sr_0.2_NiO_3_ during the reduction process leads to an infinite‐layer structure and a compressed c‐lattice parameter, which is more evident in the shift of the diffraction peak of Nd_0.8_Sr_0.2_NiO_2_ to a higher angle, and saturated at the value of 2*θ* (c = 3.36 Å), indicating the successful transition of the perovskite phase to an infinite‐layer structure (see Figure 2b). To obtain more detailed structural information, we measured the reciprocal space mapping (RSM) near the SrTiO_3_ (103) reflection as shown in Figure S1 (Supporting Information). Before and after reduction, the film is always confined to the in‐plane SrTiO_3_ lattice, indicating that the film is coherently strained by the substrate. In addition, the reciprocal space maps surrounding the (103) SrTiO_3_ diffraction peak reveal no indications of film orientation deviation.

At first glance, the high‐quality Nd_0.8_Sr_0.2_NiO_3_ phase appears to be maintained consistently throughout the cycle, demonstrating its robust stability. Yet, more careful inspection indicates that after cycle 4, it is clear that the intensity of the XRD (001) diffraction peaks of the films gradually decreased as the number of reversible cycles increased. In addition, Figure 2c demonstrates that the Bragg diffraction angle of perovskite nickelate Nd_0.8_Sr_0.2_NiO_3_ (002) film peak is gradually shifted to a smaller angle, which may be attributed to the perovskite Nd_0.8_Sr_0.2_NiO_3_ slight degradation and gradual evolution of RP‐type phase, i.e., (110) phase of (Nd_0.8_Sr_0.2_)_4_Ni_3_O_10_.^[^ ^35^ ^]^ Our density functional theory (DFT) calculations also indicate that the RP phase is more stable than the perovskite phase for nickelates (see Figure S2 and the related discussions, Supporting Information). It has also been reported that the diffraction peaks of perovskite nickelate Nd_0.8_Sr_0.2_NiO_3_ films were weakened and the *c*‐axis lattice constant increased, accompanied by the absence of Ni cations. This indicates the presence of a Ruddlesden–Popper phase.^[^ ^36^, ^37^, ^38^ ^]^ Therefore, we fitted the XRD spectrum using pristine perovskite nickelate films and a portion of the secondary phase in the film, to more clearly highlight the change in composition in the film during reversible cycling, as shown in Figure 2d, with the detailed fitted curve data shown in Figure S3 (Supporting Information). As the number of cycling experiments increases, the fitting results indicate a decrease in the pure perovskite Nd_0.8_Sr_0.2_NiO_3_ film component and a progressive increase in the RP‐type phase (Nd_0.8_Sr_0.2_)_4_Ni_3_O_10_ component in the film. Figure S4 (Supporting Information) illustrates the various phases in film during redox cycles. What's more, we complemented the cross‐sectional scanning transmission electron microscopy (STEM) images of the partial films during the reversible cyclic phase transition, where we clearly observed the presence of the RP‐type phases (Figure S5, Supporting Information). In particular, we found that the cross‐sectional STEM images of the mixed‐phase films after reduction show the segregation of two competing phases. The infinite‐layer phase is stabilized near the substrate, and the secondary phase is located roughly above the infinite‐layer phase. It can be clearly observed that with an increase in the number of oxidation–reduction cycles, the RP‐type defect phase in the infinite‐layer film gradually increases. Notably, during the subsequent topotactic reduction, the perovskite portion of the film Nd_0.8_Sr_0.2_NiO_3_ is transformed into an infinite layer structure Nd_0.8_Sr_0.2_NiO_2_ with a larger Bragg diffraction angle, while the RP‐type phase (Nd_0.8_Sr_0.2_)_4_Ni_3_O_10_ (110) is converted into a reduced RP‐type phase (Nd_0.8_Sr_0.2_)_4_Ni_3_O_8_ (100) with the lattice parameter coincides with the SrTiO_3_ (002) peak.^[^ ^35^ ^]^

Interestingly, during the number of redox cycles, even with the existence of defective RP‐type phases, the film quality of the perovskite portion after oxidation or the infinite‐layer portion after reduction seems to be consistently high. This is evidenced by the XRD rocking curve measurement for (002) diffraction peak in Figure 2e,f, where both perovskite Nd_0.8_Sr_0.2_NiO_3_ and infinite‐layer Nd_0.8_Sr_0.2_NiO_2_ exhibit essentially same full‐width‐at‐half‐maximum (FWHM) values as the pristine and first reduction. Moreover, the FWHM values for both Nd_0.8_Sr_0.2_NiO_3_ and Nd_0.8_Sr_0.2_NiO_2_ maintain nearly unbroadened with an increase of cycle numbers. This reflects that although the redox cycle can promote more secondary phases and the component of perovskite or infinite‐layer phases is indeed decreased, yet, as long as existed, the crystal quality of the remaining portions of perovskite or infinite‐layer phases remains unchanged. Combining the phase evolution data in Figure 2d–f, it is reasonable to conclude that while perovskite Nd_0.8_Sr_0.2_NiO_3_ is unstable and redox cycles facilitate the conversion from Nd_0.8_Sr_0.2_NiO_3_ to RP‐type phases (Nd_0.8_Sr_0.2_)_4_Ni_3_O_10_, however, the superconductivity of the infinite‐layer Nd_0.8_Sr_0.2_NiO_2_ is stable and can withstand several redox cycles. As long as there is a perovskite Nd_0.8_Sr_0.2_NiO_3_ phase in the film, it will be reduced into an infinite‐layer Nd_0.8_Sr_0.2_NiO_2_. This result is surprising as it is generally believed that the infinite‐layer structure of nickelate is in a metastable state, yet our data suggest that it is very stable even with ultralow valence Ni^1+^ ion. Therefore, the fundamental obstacle to the further development of nickel‐based superconductivity is still the limitation of the original crystal quality of the perovskite Nd_0.8_Sr_0.2_NiO_3_ phase.

In addition, we also calculated the variation curves of the lattice constants of the nickelate films with redox cycling experiments for both structures using the Bragg formula, as shown in Figure 2g. Furthermore, from real space atomic force microscopy (AFM) images (Figure S6, Supporting Information), we obtained films with surfaces showing atomically smooth step‐like structures (pristine), further confirming the growth of high‐quality perovskite films. Notably, the surface roughness of the films increased after the samples were subjected to multiple oxidations and reductions. Furthermore, we used X‐ray reflectivity (XRR) to confirm that the film thicknesses (see Figure 2h) of both structures exhibit stable macroscopic variation during the redox cycling experiments and the infinite‐layer structure is thinner (Figure S7, Supporting Information).

**Figure 3:**
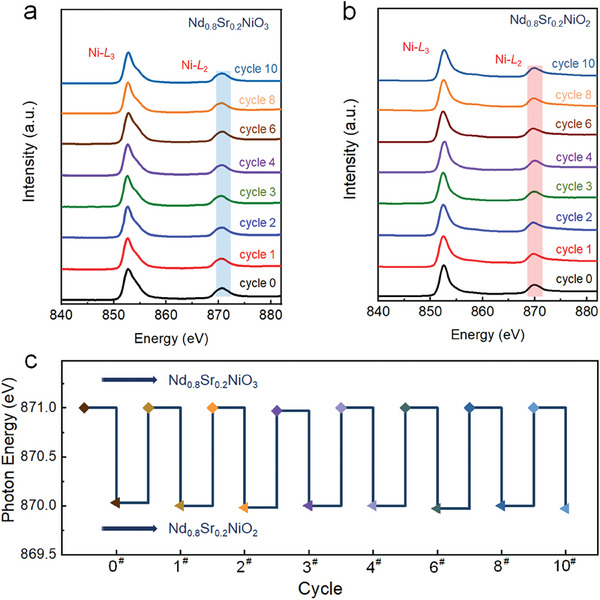
Electronic evolution of epitaxial thin film perovskite Nd_0.8_Sr_0.2_NiO_3_ growing on SrTiO_3_ (001) orientation to infinite‐layer Nd_0.8_Sr_0.2_NiO_2_ phase. a) (Color online) XAS spectra near the Ni‐*L*_2,3_ edge (3*d* state) of nickelates Nd_0.8_Sr_0.2_NiO_3_/SrTiO_3_ and b) Nd_0.8_Sr_0.2_NiO_2_/SrTiO_3_ heterostructures during the reversible cycling. c) Energy change curves of Ni‐*L*_2_ edge (3*d* state) during reversible cycling for two structures of nickelate films.

In order to understand the electronic structure evolution of nickelate films during reversible cycling experiments, ex situ XAS measurements were performed for various samples. **Figure** **3**a shows the XAS spectra of Nd_0.8_Sr_0.2_NiO_3_, where a shoulder can be seen at ≈854 eV corresponding to the transition of electrons from the Ni core level 2𝑝 to the 3𝑑^8^ *L* state (*L* is the ligand hole), beside the main sharp peak at ≈852.5 eV corresponding to the electron transition from the core‐level 2𝑝 to 3𝑑^7^ state. As the reduction of the films from perovskite to infinite‐layer structure, the prominent front peak in the Nd_0.8_Sr_0.2_NiO_2_ films disappears, which is consistent with the previously studied underdoped RNiO_2_ (R = La, Nd).^[^ ^20^, ^39^ ^]^ On the other hand, the shoulder peak of the Ni‐*L*_3_ edge spectral profile of the infinite‐layer Nd_0.8_Sr_0.2_NiO_3_ is a manifestation of the Ni 3*d*‐Nd 5*d* hybridized state, which is consistent with our recent work on doped (Nd, Sr)NiO_2_H_x_ and another work on parent NdNiO_2_ films (non‐capped).^[^ ^33^ ^]^ In addition, the Ni‐*L*_2_ absorption edge energy of the Nd_0.8_Sr_0.2_NiO_2_ film is lower than that of the Ni‐*L*_2_ absorption edge energy of the Nd_0.8_Sr_0.2_NiO_3_ film by ≈0.9 eV (Figure 3b). The observed spectroscopic features for films during redox cycles are generally with previously reported data.^[^ ^20^, ^23^, ^40^, ^41^ ^]^ Figure 3c summarizes the variation of Ni‐*L*_2_ edge energy during the reversible transition of the superconducting film with an infinite‐layer and the perovskite precursor film. Consequently, the above characterization can be demonstrated to show that the oxidation state of Ni ions in the perovskite structural framework can be reversibly controlled between trivalent and monovalent. Moreover, Figure S8a,b (Supporting Information) shows the O K‐edge XAS spectra of Nd_0.8_Sr_0.2_NiO_3_ and Nd_0.8_Sr_0.2_NiO_2_ during reversible cycling experiments, where the leading edge is clearly visible in Nd_0.8_Sr_0.2_NiO_3_ at 527.6 eV due to the charge transfer between the transition metal and ligand states.^[^ ^42^ ^]^ In addition, the pre‐edge intensity of Nd_0.8_Sr_0.2_NiO_3_ is gradually weakened as the cycling number goes on, which was attributed to the increase of RP‐type phase content and decrease of Nd_0.8_Sr_0.2_NiO_3_ phase in the film, consistent with the XRD results. When the perovskite Nd_0.8_Sr_0.2_NiO_3_ is reduced to the infinite‐layer Nd_0.8_Sr_0.2_NiO_2_ phase, the pre‐edge feature disappears, which is well‐consistent with other infinite‐layer nickelates.^[^ ^43^, ^44^ ^]^

In previous reports, the stable reduction of perovskite to infinite‐layer relied on SrTiO_3_ capping,^[^ ^9^, ^18^ ^]^ but subsequent reports showed that the coverings were not necessary.^[^ ^13^, ^20^, ^45^ ^]^ Our present work is to further demonstrate the stable superconductivity properties even after the reversible release and doping of oxygen ions in the perovskite structural framework. Here, we plotted the temperature‐dependent resistivity *ρ*(*T*) of perovskite Nd_0.8_Sr_0.2_NiO_3_ and infinite‐layer Nd_0.8_Sr_0.2_NiO_2_ films during the reversible cycling experiments.

**Figure 4:**
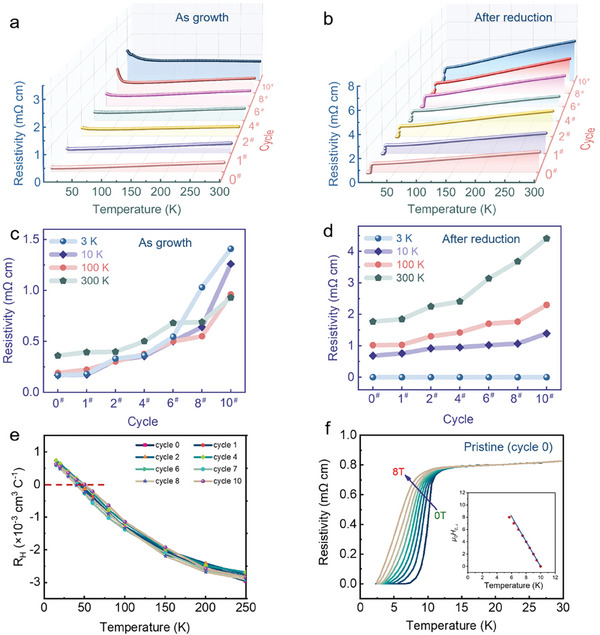
Transport properties of nickelate films. a) Temperature‐dependent resistivity measured for thin film samples of nickelates (as growth films) and b) nickelates (after reduction films) during reversible cycling. c) Variation of resistivity of as growth films and d) after reduction films at (10, 100, 300 K) during reversible cycling. e) The infinite‐layer of nickelates Nd_0.8_Sr_0.2_NiO_2_ normal‐state Hall coefficient *R*_H_ (T) corresponding to (a). f) Resistivity of superconducting infinite‐layer of nickelates Nd_0.8_Sr_0.2_NiO_2_ (pristine‐cycle 0) as a function of temperature in a varying magnetic field perpendicular to the a–b plane. The inset shows the upper critical field *H_c_*_,⊥_ as a function of temperature (estimated from the midpoint of the superconducting state transition temperature), with a linear fit around *T*_c_.

The resistivity of Nd_0.8_Sr_0.2_NiO_2_ in the high‐temperature region (*T*>100 K) in the normal state shows a significant linear relationship with temperature, similar to the temperature‐dependent resistivity of high‐temperature superconducting YBa_2_Cu_3_O_7_ films.^[^ ^46^ ^]^ The linear temperature dependence deviates at low temperatures, with the resistivity plateau initiating at ≈50 K. Finally, the resistivity plateau transforms into a superconducting phase transition from *T*_c_ with good overall conductivity and zero resistance for all redox cyclic processes, the superconducting transition onset temperature *T_c_*_90%_ (the temperature at which the resistivity drops to 90% at 15 K) is 11.5 K. The superconductive state occurs at *T*_c Zero_ ≈8 K (**Figure** **4**b). The superconducting transition temperature is compatible with the previously reported values.^[^ ^9^, ^30^ ^]^ On the other hand, the resistivity of the pristine perovskite Nd_0.8_Sr_0.2_NiO_3_ (cycle 0) films exhibited metallic behavior throughout the temperature interval (Figure 4a), which is consistent with that reported in the literature.^[^ ^9^ ^]^ However, with redox cycles, the film shows increased resistivity, especially at low temperatures, due to the presence of a pronounced RP‐type phase, especially after cycle 4, as shown in Figure 4c. Interestingly, the normal‐state resistivity of the reduced film also shows a similar increasing trend after four cycles (Figure 4d). The results indicate that the increase in overall resistivity of reduction film is due to the presence of pronounced RP‐type phases during reoxidation. Surprisingly, below *T*_c_ the reduced film always exhibits zero resistance, indicating the superconducting infinite‐layer Nd_0.8_Sr_0.2_NiO_2_ is structurally stable even after redo cycles, consistent with the XRD and XAS results. More importantly, the Nd_0.8_Sr_0.2_NiO_2_ without SrTiO_3_ capping layer exhibits stable superconductivity reproducibility and little variation in superconductivity transition temperature *T*_c_ in all ten redox cycles, which has unlimited potential for future device applications.

Figure 4e shows the temperature dependence of the normal‐state Hall coefficient *R*_H_ during several cycling experiments for Nd_0.8_Sr_0.2_NiO_2_ films, which show significant sign change from negative to positive *R*_H_ as the temperature decreases. At high temperatures, the *R*_H_ has a negative sign for all samples and is positive at low temperatures (below ≈100 K), which is a smooth transition process. The obtained magnetotransport properties of thin‐film Nd_0.8_Sr_0.2_NiO_2_ (pristine – cycle 0), along with magnetic field dependence of resistivity (Figure 4f) are overall consistent with previous reports with a mixed carrier contribution of electrons and holes.^[^ ^12^ ^]^ However, it is surprising that after ten redox cycles, the infinite‐layer nickelate *R*_H_ still exhibits a mixed carrier type of electrons and holes, with no significant changes observed. The results indicate that infinite‐layer nickelate superconductivity can be very stable and sustainable at ambient conditions even after ten redox cycles.

## 3 Conclusion

We have performed a series of redox cycling experiments on nickelate superconductors with optimal Sr doping levels and systematically investigated the structural, electronic, and transport properties of the samples across the reversible phase transition between perovskite Nd_0.8_Sr_0.2_NiO_3_ and infinite‐layer Nd_0.8_Sr_0.2_NiO_2_. It is found that the infinite‐layer Nd_0.8_Sr_0.2_NiO_2_ is generally robust and maintains stable superconductivity reproducibility after ten redox cycling experiments as demonstrated by X‐ray diffraction, X‐ray absorption, and Hall effect transport data. These results suggest that the structural stability of nickelate superconductivity is not restricted by the unusually low valence state of Ni^1+^ but by the quality of its perovskite precursor, in which the competition between perovskite main phase and RP‐type phases plays an important role. More importantly, the reversible change from perovskite Nd_0.8_Sr_0.2_NiO_3_ to infinite‐layer of Nd_0.8_Sr_0.2_NiO_2_ can be achieved by “deintercalation‐intercalation” the apical oxygen atoms of the oxygen octahedra in the perovskite structure without losing the structural framework and topological relationships. These results suggest that given high‐quality perovskite can be obtained, infinite‐layer nickelate superconductivity can be very stable and sustainable under environmental conditions. Our work provides important implications for potential device applications for nickelate superconductors.

## 4 Experimental methods

The SrTiO_3_ substrate with lattice orientation (001) was etched with buffered hydrofluoric acid (BHF) and annealed at 1000 °C for 2 h to achieve atomically flat TiO_2_ terminations. Prior to their deposition, the substrates were pre‐annealed for 1 h at 630 °C^[^ ^37^ ^]^ and 3 × 10^−6^ Torr oxygen partial pressure in order to obtain SrTiO_3_ (001) substrates with atomically flat surface morphology and cell steps. Further, the 17‐nm‐thick epitaxial film of perovskite Nd_0.8_Sr_0.2_NiO_3_ was grown on the TiO_2_‐terminated (001) oriented SrTiO_3_ single crystal substrate using a laser pulsed laser deposition (PLD) system. (Note: it was estimated that the number of laser pulses for a thickness equivalent to ≈45 unit‐cell growth based on the film deposition rate detected by RHEED. The deposition rate based on a limited number of oscillations, further estimating the film thickness was estimated.) In the present work, a uniform rectangular laser spot of 1.0 × 3 mm in size formed by aperture imaging for ablation was used. Using a laser energy density of 1.1 J cm^−2^, the oxygen partial pressure of Nd_0.8_Sr_0.2_NiO_3_ during deposition was 200 mTorr, and the substrate temperature was kept at 600 °C. The target was ablated using a laser fluence of 1.1 J cm^−2^ with a frequency of 4 Hz. All samples mentioned in the text were taken without introducing a capping layer.

In order to achieve a nickelate phase with an infinite‐layer structure, the reduction process used CaH_2_ as a reducing agent and the detailed experimental approach followed with the previous work.^[^ ^45^, ^47^ ^]^ The reduced nickelates with infinite‐layer structure were further reverse oxidized to nickelates with perovskite structure, and the samples were heated in air and annealed at 600 °C for 1 h with a temperature rise and fall rate of 10 °C min^−1^. The above redox process was repeated ten times to systematically study the physical properties of Nd_0.8_Sr_0.2_NiO_2_ after ten times of “deintercalation–intercalation” cycles.

The crystal structure of the films was characterized by high‐resolution X‐ray diffraction (XRD) and reciprocal space mapping (RSM) using a Bruker D8 Discover diffractometer. Film thickness was determined by X‐ray reflectivity (XRR) measurements from film/substrate model simulations. The surface morphology and step‐terrace structure of the grown nickelate films were characterized by atomic force microscopy (AFM, Park system, NX10). The X‐ray absorption spectroscopy (XAS) tests were performed at the Singapore Synchrotron Light Source (SSLS). All measurements were performed at 20 K, and the grazing incidence angle of the collected signal was *θ* = 20°. The fluorescence yield XAS spectra were collected with a photodiode and normalized to the incident beam intensity. The resistivity of the films was obtained by a four‐probe method using aluminum wire bonding contacts in a cryogenic magnet‐free system (CFMS, Cryogenic) with temperature‐dependent resistivity and Hall effect.

## Conflict of Interest

The authors declare no conflict of interest.

## Author contributions

M.X. and Y. Z. contributed equally to this work. L.Q. conceived the idea and supervised the project. M.H.X. and Y.Z. synthesized the perovskite nickelate thin films and performed the topotactic reduction experiments. M.H.X. and X.D. characterized the crystalline structure. Z.H. and M.S.L. performed the TEM measurements. M.H.X. and Y.Z., performed the transport measurements with the help of H.Q.L., X. Q. W., and X.D. M.H.X. and Y.Z. analyzed the transported data. J. B. Y., X. J. Y., M. B.H. B., and S. B. X., performed XAS measurements. M.H.X., Y.Z., and L.Q. wrote the manuscript with input from all the authors.

## Supporting information

Supporting Information

## Data Availability

The data that support the findings of this study are available from the corresponding author upon reasonable request.
