# Supplementary material for: Robust Superconductivity in Infinite‐Layer Nickelates
Source: Adv Sci (Weinh). 2024 Apr 29;11(37):2305252. doi: 10.1002/advs.202305252 (PMC11462288; doi:10.1002/advs.202305252)
Supplement: Supplementary file 1 — Supporting Information [file ADVS-11-2305252-s001.pdf]

ADVANCED SCIENCE
Open Access

Supporting Information

for *Adv. Sci.*, DOI 10.1002/advs.202305252

Robust Superconductivity in Infinite-Layer Nickelates

Minghui Xu, Yan Zhao, Yu Chen, Xiang Ding, Huaqian Leng, Zheng Hu, Xiaoqiang Wu, Jiabao Yi, Xiaojiang Yu, Mark B.H. Breese, Shibo Xi, Mengsha Li* and Liang Qiao**

Robust Superconductivity in Infinite-layer Nickelates

Minghui Xu, Yan Zhao, Yu Chen, Xiang Ding, Huaqian Leng, Zheng Hu, Xiaoqiang Wu, Jiabao Yi, Xiaojiang Yu, Mark B.H. Breese, Shibo Xi, Mengsha Li, and Liang Qiao**

M. H. Xu, Y. Zhao, Y. Chen, X. Ding, H. Q. Leng, and L. Qiao
School of Physics
University of Electronic Science and Technology of China
Chengdu 610054, China
Email: liang.qiao@uestc.edu.cn
Z. Hu, M. S. Li
Center for Microscopy and Analysis
Nanjing University of Aeronautics and Astronautics
Nanjing 211100, China
Email: limengsha@nuaa.edu.cn
X. Q. Wu
Institute for Advanced Study
Chengdu University
Chengdu 610106, China.
J. B. Yi
Global Innovative Centre for Advanced Nanomaterials, School of Engineering
The University of Newcastle
Callaghan, NSW, 2308, Australia.
X. J Yu, M. B.H. Breese, S. B. Xi
Singapore Synchrotron Light Source
National University of Singapore
Singapore, 117603, Singapore

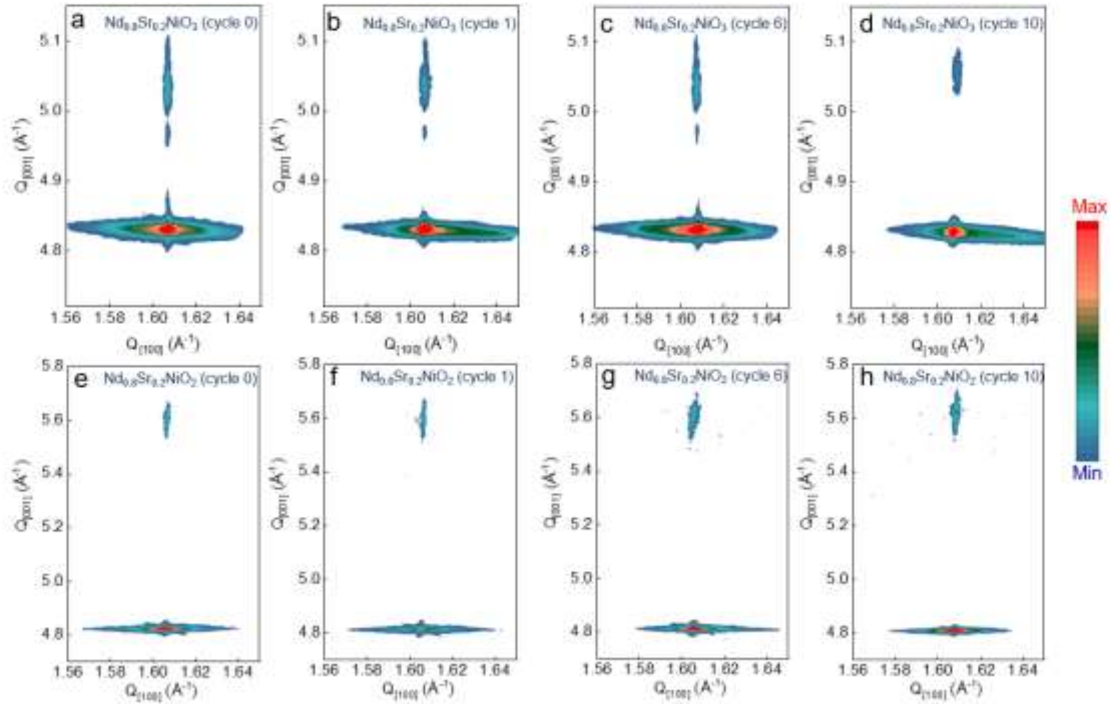

Figure S1. Reciprocal Space Mapping of the (103) diffraction peak from the pristine Cycle 0 (primary), Cycle 1, Cycle 6, and Cycle 10 samples. Note that the RSM data yield the same c-axis lattice constants as retrieved from the XRD data.

Previous theoretical studies have suggested that chemical potential plays an important role in terms of oxides' thermodynamic stability. To investigate the thermodynamic stability of $NdNiO_3$, we perform the DFT calculations. To avoid the formation of possible secondary phases, such as, Nd_2NiO_4, $Nd_3Ni_2O_7$, and $Nd_4Ni_3O_{10}$, the following relations should be required:

$$2\mu_{Nd} + \mu_{Ni} + 4\mu_O < \Delta H_f(Nd_2NiO_4), \quad (R1)$$

$$3\mu_{Nd} + 2\mu_{Ni} + 7\mu_O < \Delta H_f(Nd_3Ni_2O_7), \quad (R2)$$

$$4\mu_{Nd} + 3\mu_{Ni} + 10\mu_O < \Delta H_f(Nd_4Ni_3O_{10}), \quad (R3)$$

where μ_{Nd}, μ_{Ni}, and μ_O indicate the chemical potential of Nd, Ni, and O elements, as well as $\Delta H_f(Nd_2NiO_4)$, $\Delta H_f(Nd_3Ni_2O_7)$, and $\Delta H_f(Nd_4Ni_3O_{10})$ indicate the formation enthalpy of Nd_2NiO_4, $Nd_3Ni_2O_7$, and $Nd_4Ni_3O_{10}$, respectively. On the other hand, to restrain the formation of elementary substances and maintain the stable phase of $NdNiO_3$, μ_{Nd}, μ_{Ni}, and μ_O should satisfy,

$$\mu_{Nd} < 0,\ \mu_{Ni} < 0, \text{ and } \mu_O < 0, \quad (R4)$$

$$\mu_{Nd} + \mu_{Ni} + 3\mu_O = \Delta H_f(NdNiO_3). \quad (R5)$$

Taking all constraints into account, the achievable chemical potential region for $NdNiO_3$ is plotted in **Figure R4**. It is clear that the stable region of $NdNiO_3$ is limited in a narrow range and most areas are occupied by possible secondary phases. This implies chemical potential μ_{Nd}, μ_{Ni}, and μ_O should be restricted in certain regions, otherwise, the secondary phase would be created. Our results suggest the oxygen chemical potential ($\mu_O = [\Delta H(NdNiO_3)-\mu_{Nd}-\mu_{Ni}]/3$) should be strictly limited to -1.012~0 eV, and if it is less than -1.012 eV (corresponding to a lower oxygen pressure experimentally), secondary phases would be introduced. Therefore, this result suggest that element chemical potentials should be carefully controlled for the purpose of suppressing the formation of RP phases.

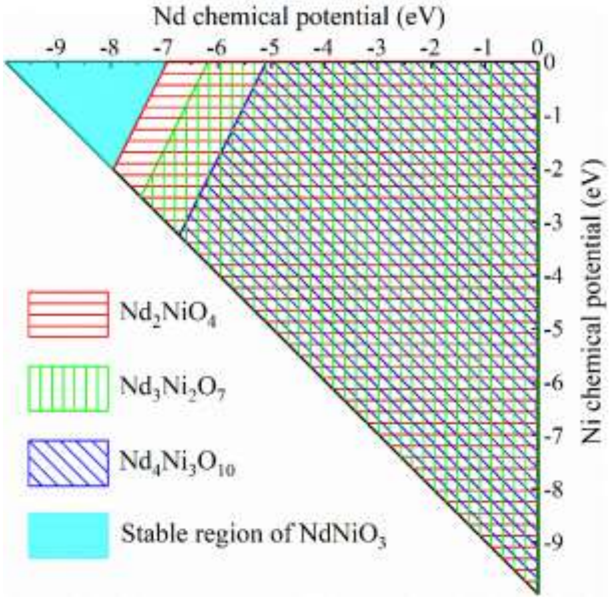

Figure S2. Simulated thermodynamic stability phase diagram of $NdNiO_3$. The cyan shadow indicates the stable region of $NdNiO_3$ and possible secondary phases (RP phase) including Nd_2NiO_4, $Nd_3Ni_2O_7$, and $Nd_4Ni_3O_{10}$ are shown in grid regions with different colors.

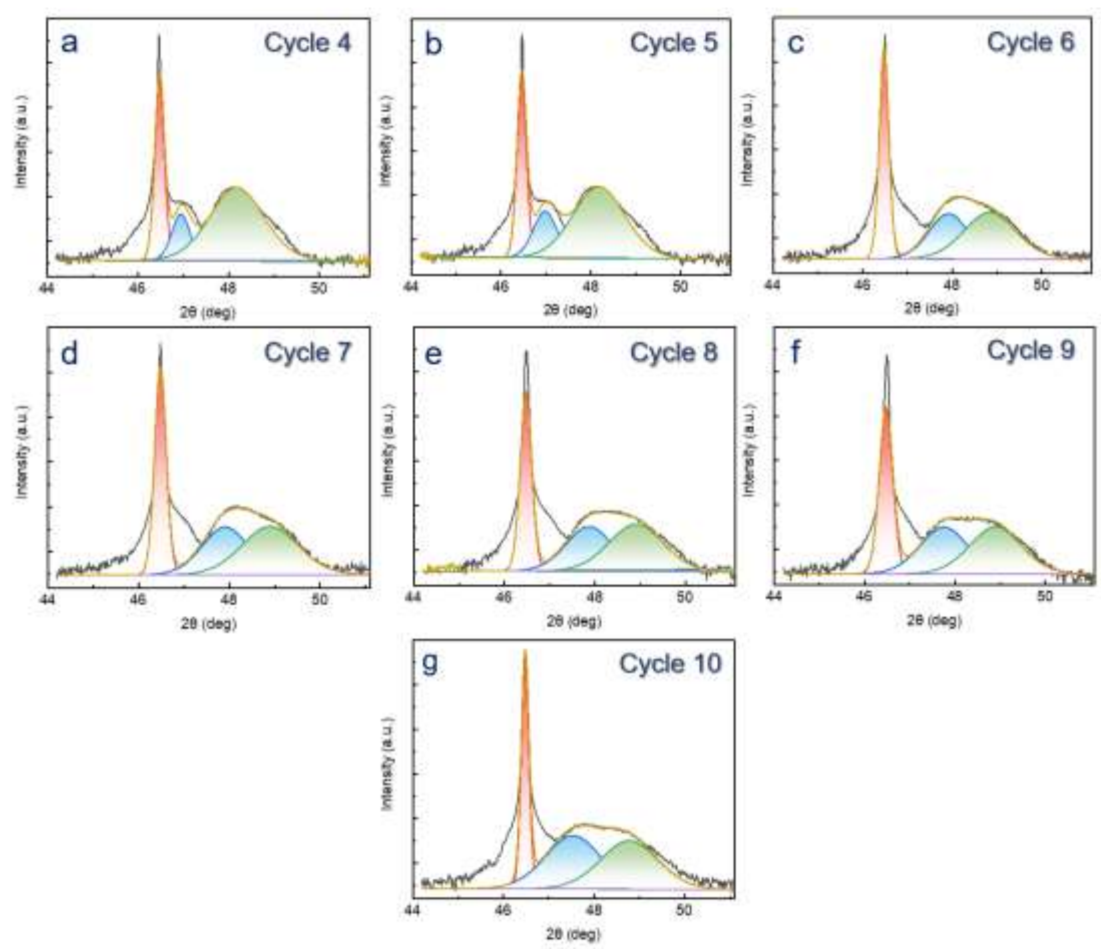

Figure S3. Structural competition between the generation of Ruddlesden-Popper defect phase and perovskite $Nd_{0.8}Sr_{0.2}NiO_3$ during the reversible transition, respectively, is the relationship between the (002) diffraction peaks fitted to the perovskite precursor in cycle 4-cycle10.

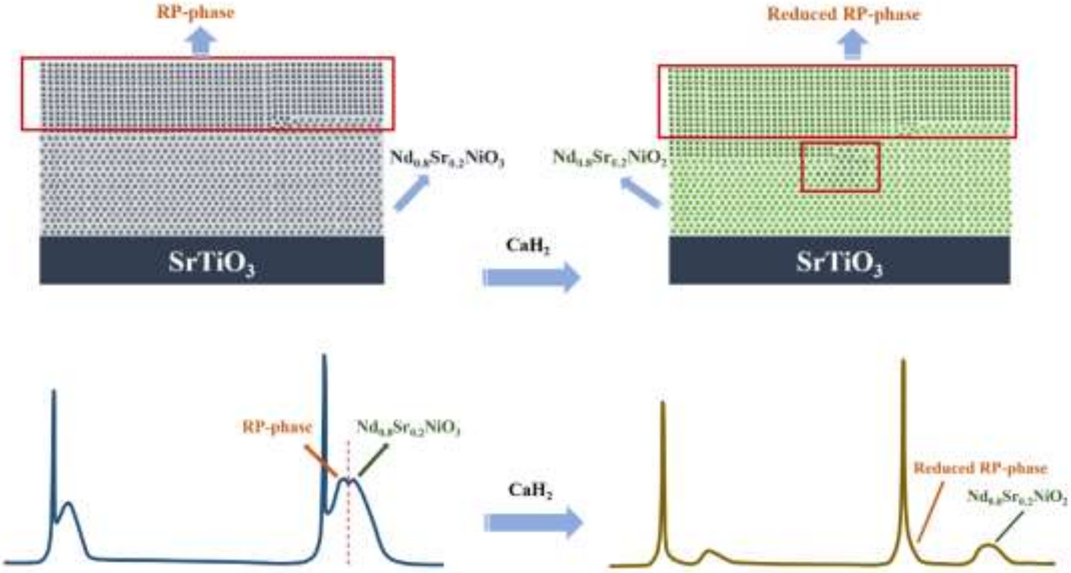

Figure S4. Schematic illustration of the reduction process of the Ruddlesden-Popper defect phase and perovskite $Nd_{0.8}Sr_{0.2}NiO_3$ during reversible experiments.

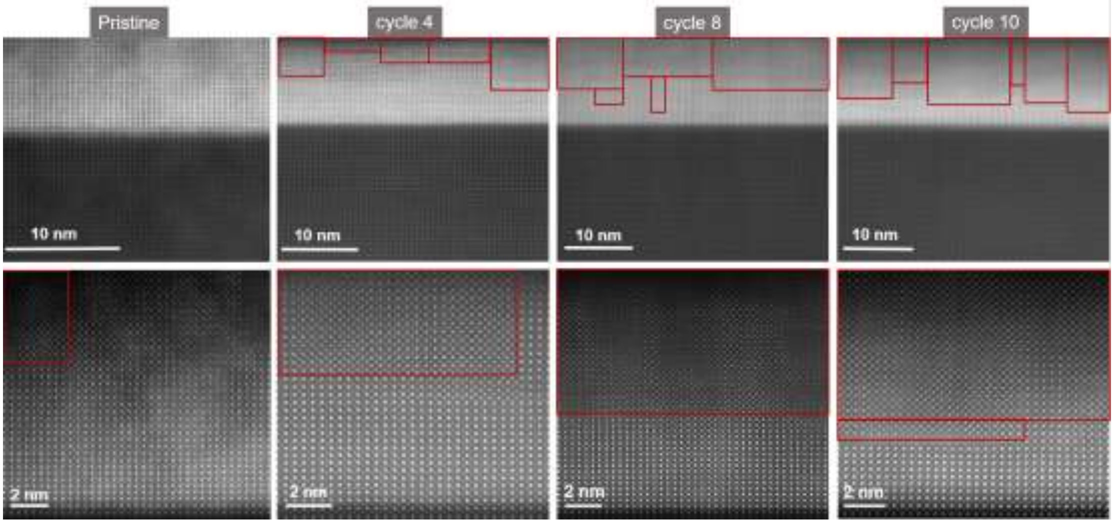

Figure S5. HAADF-STEM characterization for the superconducting $Nd_{0.8}Sr_{0.2}NiO_2$ sample during reversible cycling. Regions bounded by Ruddlesden-Popper type stacking faults are outlined in red lines. (Pristine, cycle 4, cycle 8 and cycle 10)

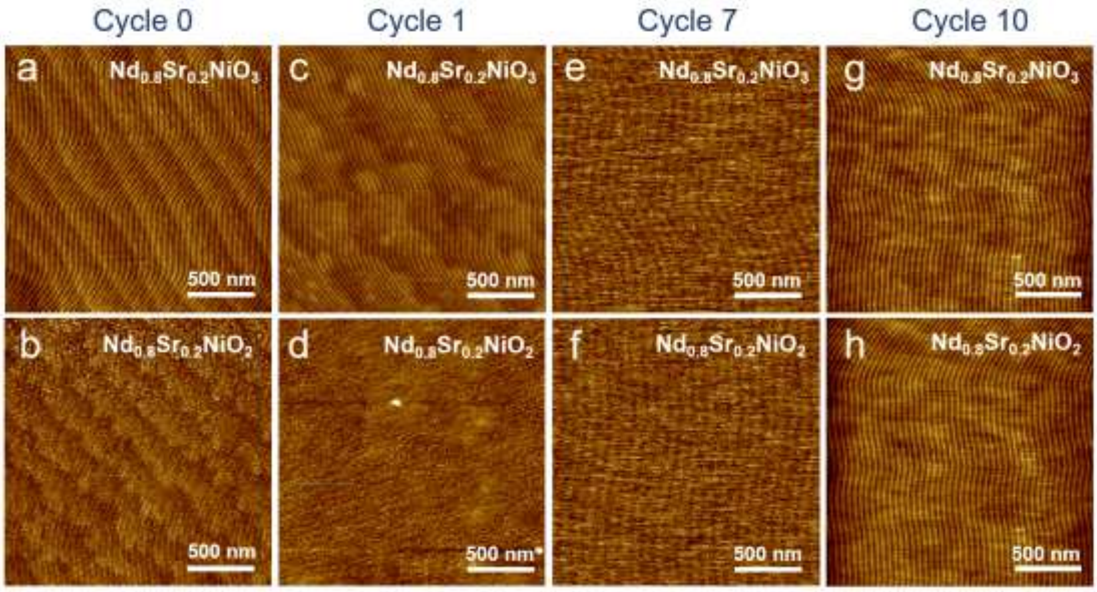

Figure S6. AFM images of the pristine Cycle 0 (primary), Cycle 1, Cycle 6, and Cycle 10 samples. The AFM images (Cycle 0) show the root mean square of the average height deviation (R_q) in the order of 0.1 nm demonstrating a good surface quality.

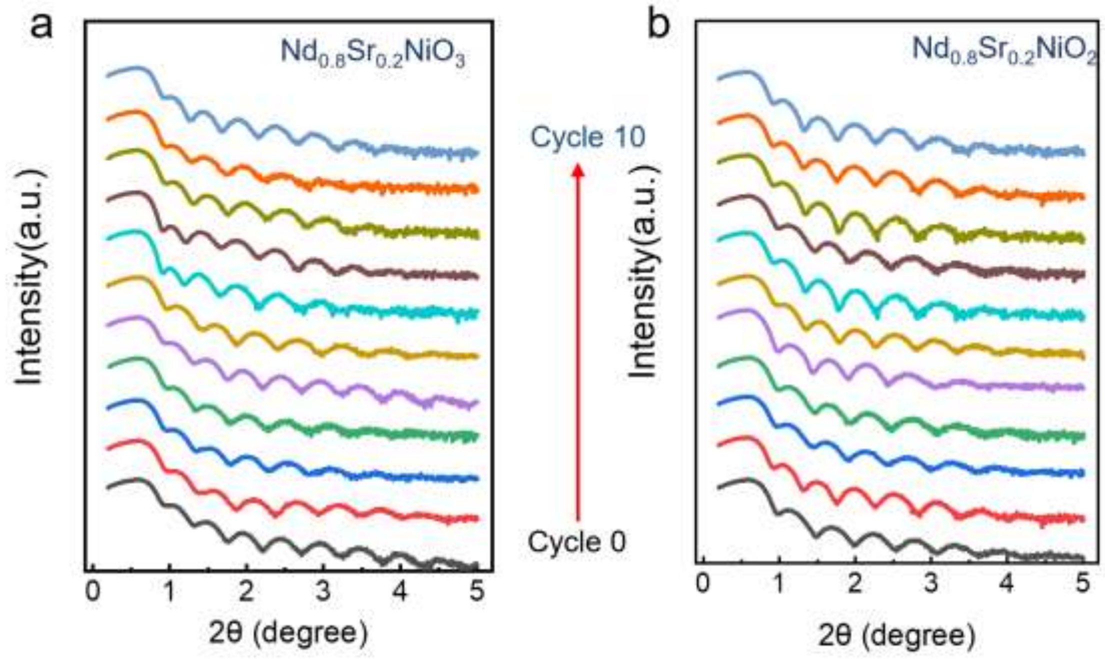

Figure S7. (a) X-ray reflectance of the nickelate $Nd_{0.8}Sr_{0.2}NiO_3/SrTiO_3$ and (b) $Nd_{0.8}Sr_{0.2}NiO_2/SrTiO_3$ heterostructures during the reversible cycling.

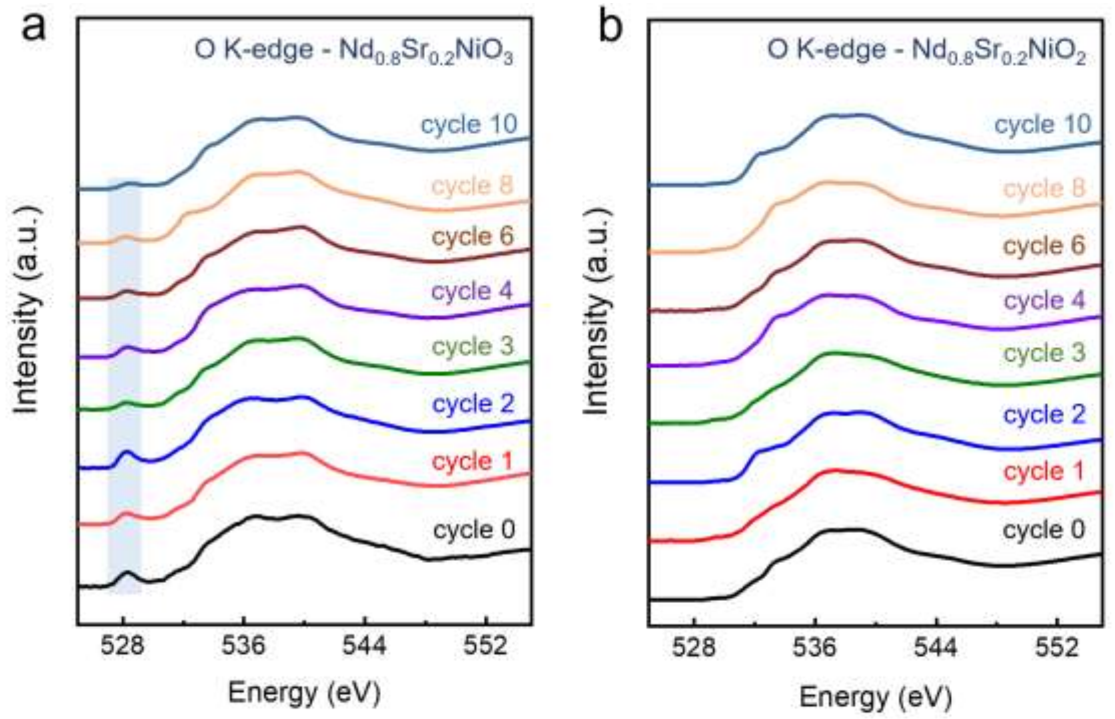

Figure S8. (a) XAS spectra near the O-K edge of nickelates $Nd_{0.8}Sr_{0.2}NiO_3/SrTiO_3$ and (b) $Nd_{0.8}Sr_{0.2}NiO_2/SrTiO_3$ heterostructures during the reversible cycling.
